# Prevalence of microvascular angina among patients with stable symptoms in the absence of obstructive coronary artery disease: a systematic review

**DOI:** 10.1093/cvr/cvab061

**Published:** 2021-03-02

**Authors:** Elif Aribas, Jeanine E Roeters van Lennep, Suzette E Elias-Smale, Jan J Piek, Maurits Roos, Fariba Ahmadizar, Banafsheh Arshi, Dirk J Duncker, Yolande Appelman, Maryam Kavousi

**Affiliations:** 1 Department of Epidemiology, Erasmus MC, University Medical Center Rotterdam, Office Na-2714, PO. Box 2040, 3000 CA Rotterdam, The Netherlands; 2 Department of Internal Medicine, Erasmus MC, University Medical Center, Rotterdam, The Netherlands; 3 Department of Cardiology, Radboud University Medical Center, Nijmegen, The Netherlands; 4 Department of Cardiology, Amsterdam University Medical Centers, location AMC, Amsterdam, The Netherlands; 5 Department of Cardiology, Erasmus MC, University Medical Center Rotterdam, Rotterdam, The Netherlands; 6 Department of Cardiology, Amsterdam University Medical Centers, Location VU University Medical Center, Amsterdam, The Netherlands

**Keywords:** Microvascular angina, Coronary microvascular dysfunction, Prevalence, Systematic review, Non-obstructive coronary artery disease

## Abstract

Our purpose was to perform a systematic review to assess the prevalence of microvascular angina (MVA) among patients with stable symptoms in the absence of obstructive coronary artery disease (CAD). We performed a systematic review of the literature to group the prevalence of MVA, based on diagnostic pathways and modalities. We defined MVA using three definitions: (i) suspected MVA using non-invasive ischaemia tests; proportion of patients with non-obstructive CAD among patients with symptoms and a positive non-invasive ischaemia test result, (ii) suspected MVA using specific modalities for MVA; proportion of patients with evidence of impaired microvascular function among patients with symptoms and non-obstructive CAD, and (iii) definitive MVA; proportion of patients with positive ischaemia test results among patients with an objectified impaired microvascular dysfunction. We further examined the ratio of women-to-men for the different groups. Of the 4547 abstracts, 20 studies reported data on MVA prevalence. The median prevalence was 43% for suspected MVA using non-invasive ischaemia test, 28% for suspected MVA using specific modalities for MVA, and 30% for definitive MVA. Overall, more women were included in the studies reporting sex-specific data. The women-to-men ratio for included participants was 1.29. However, the average women-to-men ratio for the MVA cases was 2.50. In patients with stable symptoms of ischaemia in the absence of CAD, the prevalences of suspected and definitive MVA are substantial. The results of this study should warrant cardiologists to support, promote and facilitate the comprehensive evaluation of the coronary microcirculation for all patients with symptoms and non-obstructive CAD.

## 1. Introduction

Although the focus in ischaemic heart disease is still on obstructive coronary artery disease (CAD), pathological conditions of the coronary microvessels, including abnormal vasodilatory responses or spasm of the microvessels, are gaining more attention as they appear to be important causes of angina and myocardial ischaemia when no obstructive CAD is found on the diagnostic coronary angiogram. Although the traditional cardiovascular risk factors are identified as important modifiable risk factors for this disorder, the underlying pathophysiological mechanisms remains to be elucidated.^1^^,^^2^

Patients with this so-called ‘microvascular angina’ (MVA) have an increased risk of major cardiovascular adverse events including cardiovascular mortality.^3^ Furthermore, since MVA is still often overlooked in daily cardiology practice, persistent symptoms frequently lead to subsequent repeated coronary angiograms, emergency room visits, hospitalizations, and reduced quality of life.^4^ As such, adequate and accurate diagnosis of patients with MVA is of importance to initiate timely appropriate treatment, aiming to decrease morbidity and mortality risks associated with this disorder.

The diagnosis of MVA can be difficult. Different types of diagnostic modalities include non-invasive ischaemia testing or modalities to diagnose impaired microvascular function [including impaired coronary flow reserve (CFR), coronary microvascular spasm, indices of abnormal coronary microvascular resistance, and coronary slow flow phenomenon] or a combination of both.^5^ Several studies have reported a high prevalence of MVA in patients with signs and symptoms of ischaemia and no obstructive CAD.^6^^,^^7^ However, definitions of MVA differed between the various studies as a uniform definition was lacking. Recently, for the first time, international standardized criteria for MVA were published by the Coronary Vasomotion Disorders International Study (COVADIS) Group, presenting two types of MVA; suspected MVA and definitive MVA.^5^

To our knowledge, no previous systematic reviews or systematic review have addressed the prevalence of MVA among patients with stable symptoms and documented non-obstructive CAD or a positive ischaemia test result. We performed a systematic review of the literature to group the prevalence of MVA, based on diagnostic pathways and modalities. Next, we compared how these groups conform with the recently introduced COVADIS categories.

## 2. Methods

### 2.1 Definitions

The definitions were based on three levels of information regarding diagnostic pathways and modalities including; presence of stable symptoms, non-obstructive CAD, and a non-invasive stress test result and/or (non)-invasive test result for microvascular dysfunction.

**Figure 1:**
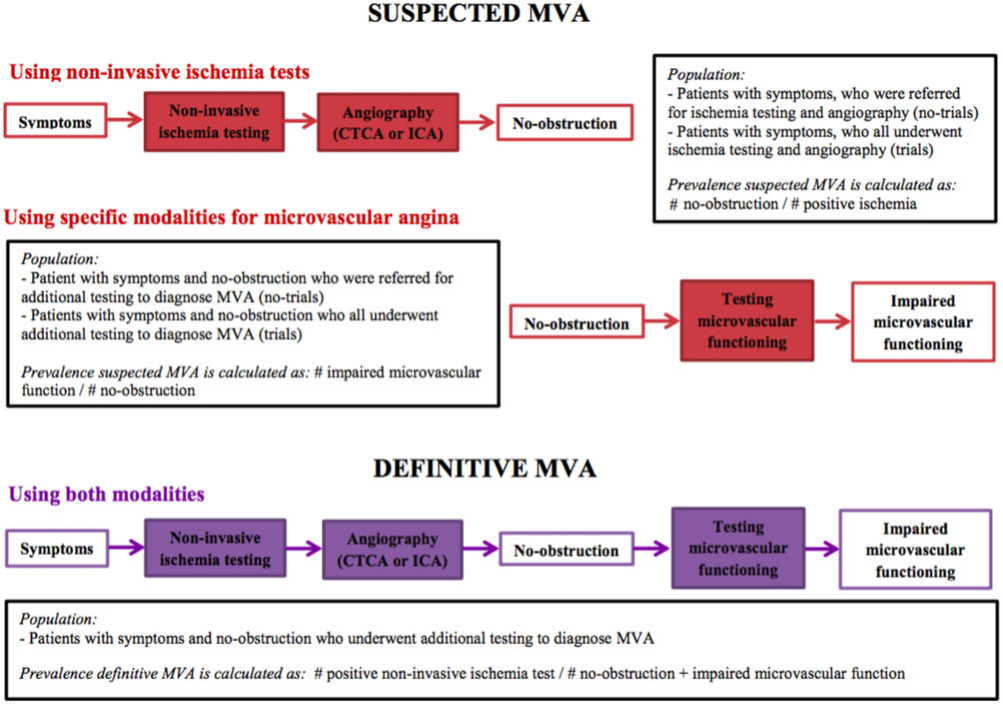
Flowchart of the diagnostic pathway of the included studies to define the prevalences for suspected and definitive MVA. MVA; microvascular angina, CTCA; computed tomography coronary angiography, ICA; invasive coronary angiography.

The following definitions were used: (i) suspected MVA using non-invasive ischaemia tests; proportion of patients with non-obstructive CAD among patients with symptoms and a positive non-invasive ischaemia tests, (ii) suspected MVA using specific modalities to diagnose MVA; proportion of patients with objective evidence of impaired microvascular function among patients with symptoms and non-obstructive CAD, and (iii) definitive MVA; proportion of patients with positive ischaemia tests among patients with an objectified impaired microvascular function. An overview of the definitions for different types of MVA and the corresponding formula for calculation of the prevalence of each type is depicted in *Figure 1*. Furthermore, no-obstruction, positive ischaemia test result, and impaired microvascular function were defined as per-individual paper (Supplementary material online, *Table S1a* and *b*).

### 2.2 Inclusion and exclusion criteria

As the primary aim of this study was to assess the prevalence of MVA, only studies fulfilling the following specific criteria for adequate study design and representative sample to retrieve prevalence data were included: (i) patients were consecutively enrolled in data registries or included in trials; (ii) number of participants ≥100. Additionally, per prevalence type, specific inclusion criteria were used regarding the study population. For prevalence of suspected MVA using non-invasive ischaemia tests, the study population needed to consist of patients with symptoms suggestive for CAD for whom a non-invasive stress test was performed and who were referred for computed tomography coronary angiography or invasive coronary angiography. For prevalence of suspected MVA using specific modalities to diagnose MVA, the study population needed to consist of patients with symptoms suggestive for CAD and without obstruction (using computed tomography coronary angiography or invasive coronary angiography) in whom additional (non)-invasive testing for microvascular dysfunction was performed. For the prevalence of definitive MVA, the population needed to consist of patients with symptoms suggestive of CAD, no-obstruction and evidence of impaired microvascular function. A flowchart visualizing the diagnostic pathways of the included studies and the study populations is depicted in *Figure 1*.

Only original studies and studies published in the English language were considered.

Studies assessing MVA in other clinical settings, including patients with known obstructive CAD or a history of obstructive CAD, defined as prior myocardial infarction, obstruction on angiography, or other cardiac morbidity were excluded. Furthermore, studies including patients with acute symptoms and/or acute coronary syndrome [myocardial infarction with non-obstructive coronary arteries (MINOCA)] were excluded (i.e. also patients with Takotsubo syndrome). No further exclusion criteria based on symptoms were used. Studies including patients with angina-like symptoms, either typical or atypical symptoms were included. Also, no restrictions were made for the duration of the symptoms (i.e. patients with chronic angina were also included).

Studies reporting data from the same study population were excluded. If study populations were overlapping, the study with the largest number of participants was included.

### 2.3 Literature search and systematic assessment of the available literature

We conducted a literature search using five electronic databases (EMBASE, Medline via Ovid, and Cochrane CENTRAL via Wiley and Google Scholar) from inception to May 2019 (date last searched). The search strategy for each database was designed by an experienced medical information specialist and consisted of terms for CAD, angiography, no-obstruction, and specific terms for the included invasive and non-invasive diagnostic modalities for diagnosis of MVA including non-invasive stress tests, acetylcholine coronary reactivity test, also called acetylcholine test, hyperaemic microvascular resistance, index of microcirculatory resistance, and TIMI frame count (TFC) and CFR measured invasive, or non-invasively by positron emission tomography, single-photon emission computed tomography, transthoracic Doppler echocardiography (TTDE), and myocardial contrast echocardiography. The full search strategy is provided as Supplementary material online, *Methods 1*. Abstract and full-text screening was performed by two independent investigators. A PRISMA checklist and flow diagram are provided as Supplementary material online, *Methods 2* and *3*).

### 2.4 Data extraction and statistical analysis

Data of the included studies were extracted independently by two investigators from the full papers. Any discrepancies between the investigators were resolved by consensus. The data retrieved included first author, publication year, study design, study population, baseline clinical characteristics, numbers, frequencies, percentages, prevalence, diagnostic modalities, definitions for a positive ischaemia test results, no-obstructive CAD, and impaired microvascular function, prognostic outcomes of patients among patients with MVA. If data were unclear or additional data were needed, authors were contacted by email. Due to expected high heterogeneity across individual studies, studies were grouped in pre-specified subgroups for analysis, including; diagnostic modality, study design (trials vs. no-trials), definitions used and sex.

## 3. Results

Of the 4547 abstracts retrieved by our search strategy, 20 studies reported data on the prevalence of MVA and were included in this study. The flowchart of the study selections is depicted in Supplementary material online, *Figure S1*. Characteristics of the included study populations are provided in the Supplementary material online, *Table S1a*–*c*).

### 3.1 Prevalence’s of suspected and definitive MVA

#### 3.1.1 Prevalence of suspected MVA using non-invasive ischaemia tests

Three trials^8–10^ and six studies^11–16^ of data registries were included with a total of 442 206 patients (44% female, mean age 60.4 years). This number consists of one large data registry contributing 423 080 patients and eight other studies, which contributed a total of 19 126 patients. The median prevalence of suspected MVA using non-invasive ischaemia tests was 43%, ranging between 33% and 65%.

Higher percentages were observed for: (i) studies that were no-trials compared to trials (median prevalence vs. median prevalence; 48% vs. 35%, respectively), (ii) studies using invasive coronary angiography compared to computed tomography coronary angiography (49% vs. 35%, respectively), (iii) studies using broader definitions of non-obstructive CAD compared to studies using the more widely used cut-off of <50% stenosis (50% vs. 43%, respectively), and (iv) studies using exercise treadmill test compared to studies using other diagnostic modalities (53% vs. 36%, respectively) (*Table 1*).

**Table 1:** Overview of the prevalence of suspected MVA using non-invasive ischaemia tests

	Total	Study design		Angiography type				Definition of NOCAD	
		Trials	No-trials	Trials, ICA	Trials, CTCA	No-trials, ICA	No-trials, CTCA	<50%	≤70%/50%^A^
*N* studies	9	3	6	1	2	3	3	7	2
Median	43%	35%	48%	44%	34%	53%	37%	43%	50%
Range	33–65%	33–44%	34–65%	44%	33–35%	43–65%	34–53%	33–53%	34–65%

CTCA, computed tomography coronary angiography; ICA, invasive coronary angiography; MVA, microvascular angina; NOCAD, no-obstructive coronary artery disease.

^A^≤70% a major epicardial coronary artery, ≤50% left main stenosis.

#### 3.1.2 Prevalence of suspected MVA using specific modalities for MVA

Eleven^6^^,^^17–26^ studies reported sufficient data to assess the prevalence of suspected MVA using specific modalities for MVA. Six studies assessed MVA by invasive acetylcholine coronary reactivity test, three studies by CFR (invasive or non-invasive) and two studies by TFC. Among the 11 studies, seven studies reported sex-specific data for both included total population and the prevalence proportion (Supplementary material online, *Table S1b*).

The median prevalence of suspected MVA using specific modalities for MVA was 27.8%, ranging between 13.5% and 46.5%. While larger prevalences were observed for studies using the TFC (median prevalence: 33.9%) and for invasive CFR count (prevalence: 46.5%, one study), lower prevalences were observed for studies using the acetylcholine test (median prevalence: 28.2%) and the TTDE (median prevalence: 25.2%).

After stratifying for type of diagnostic modality, a more narrow range was observed for studies using TFC and TTDE CFR.

After grouping for study design, a higher prevalence was observed for trials compared to non-trials (median prevalence: 34.8% vs. 26.7%) (*Table 2*).

**Table 2:** Overview of the prevalence of suspected MVA using specific modalities for MVA

	Total	Study design		Diagnostic modality			
		Trials	No-trials	Acetylcholine test	IV CFR	TTDE CFR	TFC
*N* studies	11	6	5	6	1	2	2
Median	27.8%	34.8%	26.7%	28.2%	46.5%	25.2%	33.9%
Range	13.5–46.5%	13.5–46.5%	15.2–33.2%	13.5–40.0%	46.5%	22.1–25.2%	27.8–39.9%

CFR, coronary flow reserve; IV, invasive; MVA, microvascular angina; N, number; TFC, TIMI frame count; TTDE, transthoracic Doppler echocardiography.

#### 3.1.3 Prevalence of definitive MVA

Four^6^^,^^21^^,^^23^^,^^25^ of the included studies reported data on the prevalence of definitive MVA for a total of 1380 participants with 449 cases with MVA. The median prevalence of definitive MVA was 30% (range 19.7–60.0%). Non-invasive ischaemia testing was not systematically performed in all patients. The proportions of patients that had undergone a non-invasive ischaemia test were 79.8%, 73.7%, 72.4%, and 83%, respectively. However, not all studies specified when the non-invasive ischaemia testing was performed.

The included studies used multiple different non-invasive stress tests. However, the number of performed non-invasive ischaemia tests within each study was not reported in any of the included studies (Supplementary material online, *Table S1c*).

In line with suspected MVA, a larger prevalence was observed for invasively assessed CFR (37%), while the lowest prevalence was observed for studies using the acetylcholine test (median: 24%). However, studies using multiple non-invasive ischaemia tests reported the highest prevalences (median: 49%).

### 3.2 Clinical characteristics

Among the 11 studies reporting data for the prevalence of suspected MVA using specific modalities for MVA, 7 studies (2425 patients) reported data on clinical characteristics and cardiovascular risk factors for patients with MVA and patients without MVA.

No large differences were observed for patients with MVA compared to patients without MVA regarding age (mean 62 vs. 60 years) and presence of diabetes (mean 16% vs. 15%).

However, a larger proportion of patients with MVA had hypertension (61% for MVA patients compared to 56% for patients without MVA) and were non-current smokers (78% vs. 70%). The proportion of family history of cardiovascular disease was 45% among patients with MVA and 39% among patients without MVA.

The frequency of hyperlipidaemia was 50% in both groups.

### 3.3 Sex differences

#### 3.3.1 Inclusion of patients

Among the seven studies reporting sex-specific data, one study included more men (59%), five studies included more women (57%, ranging between 53% and 69%), and one study included men and women approximately evenly. Overall, more women were included in the studies reporting sex-specific data; the women-to-men ratio for included participants was 1.29 (median 1.30).

Of the studies reporting sex-specific data, three studies were trials and four studies were non-trials. Overall, the average women-to-men ratio was 1.51 (median 1.33) in trials vs. 1.13 in no-trials (median 1.21).

#### 3.3.2 MVA cases

The average women-to-men ratio for suspected MVA using specific modalities for MVA cases was 2.50 (median 3.29). All studies reported more female cases, independent of whether these studies had included more women (six study, median female cases: 73%) or not (one study, 51% female cases). In trials, a higher women-to-men ratio for cases (median: 4.24) was reported compared to non-trials (median: 2.11). However, all trials assessed microvascular spasm using the acetylcholine coronary reactivity test.

Additionally, most studies that reported sex-specific data used the acetylcholine coronary reactivity test (*n* = 6) and only one study used TTDE CFR. No study using TFC in this study reported sex-specific data.

## 4. Discussion

Our systematic review provides a comprehensive overview of the prevalence of MVA, based on the diagnostic pathways and modalities. We reported a median prevalence of 43% for suspected MVA among patients with symptoms and a positive non-invasive ischaemia test result (suspected MVA using non-invasive ischaemia tests), a median prevalence of 28% for suspected MVA among patients with symptoms and no-obstruction (suspected MVA using specific modalities for MVA) and a median prevalence of 30% for definitive MVA among patients with symptoms, no-obstruction and evidence of impaired microvascular function. Moreover, we also showed that prevalence of suspected MVA using non-invasive ischaemia tests, among patients with a positive ischaemia test result, varies depending on study design (trial vs. no-trial), type of stress test used, type of angiography (computed tomography coronary angiography vs. invasive coronary angiography) and the definition used for no-obstructive CAD. Similarly, the prevalence of suspected MVA using specific modalities for MVA, among patients with no-obstruction and symptoms, varied depending on the choice of diagnostic modality for assessing the microvasculature. Our median prevalence of 30% for definitive MVA underlines that a large proportion of patients who present with symptoms caused by MVA will remain undiagnosed if only non-invasive ischaemia testing is used for the diagnosis.

### 4.1 Interpretation of the prevalence’s for different types of MVA

The median prevalence of suspected MVA using non-invasive ischaemia tests; the proportion of no-obstructive CAD among symptomatic patients with a positive ischaemia test result was 43%. A previous meta-analysis reported a high prevalence of 67% for no-obstructive CAD among patients with symptoms. However, regarding the prevalence of the latter study, it should be emphasized that not all patients who present with symptoms have MVA, as not all patients have had ischaemia tests prior to coronary angiography. In another clinical setting, among patients presenting with NSTEMI-ACS and myocardial infarction, only 13% [95% confidence interval (CI) 11–16%] and 6% (95% CI 5–7%), respectively were found to have no-obstructive CAD.^27^^,^^28^

In our study, the median prevalence of suspected MVA using specific modalities for MVA, the proportion of impaired microvascular functioning among patients with no-obstruction and symptoms was 28%. In the current study, we only included studies that had enrolled patients consecutively. A previous report by Sara *et al*.,^7^ assessing the prevalence of MVA among one of the largest cohorts of patients with symptoms and no obstruction in which invasive physiological testing was performed (corresponding to the definition of suspected MVA using specific modalities for MVA), was excluded for this reason. Interestingly, this study reported a twice as high percentage of suspected MVA using specific modalities for MVA, compared to the median prevalence in our study.^7^ Although both the report by Sara *et al*. and our study represents data from specialized centres performing additional physiological testing for the assessment of MVA, and therefore included a selected population, large differences in prevalence are observed. The patient population in the report by Sara *et al*. were referred for clinical testing based on the physician’s decision (so not consecutively) and therefore represent a highly selected population. This is in contrast to the ACOVA study,^6^ a prospective-trial including patients consecutively for additional testing to diagnose MVA, that reported a similar proportion as the median prevalence in our study. This shows that a selected population, referred specifically to a specialized centre, might show a higher prevalence of MVA and that prevalence in this group might be an overestimation of the prevalence in the general population of patients in daily cardiology practice.

The median prevalence of definitive MVA, the proportion of patients with positive ischaemia test result among patients with symptoms, no-obstruction and evidence of impaired microvascular function was 30%. A wide range for the prevalence of definitive MVA was observed due to use of different diagnostics modalities, number of tests performed and heterogeneity among study populations. Prior studies have shown that non-invasive ischaemia tests may not correlate with invasive microvascular function tests,^17^^,^^29^ which is in line with the reported median prevalence of 30% in our study. Therefore, when using only non-invasive ischaemia tests to diagnose patients with MVA, a large proportion of patients with impaired microvascular function might not be diagnosed as such. A previous study suggests that a large proportion of cardiologists practicing in non-academic hospitals prefer to manage patients with suspected MVA by themselves^30^ instead of referring them to specialized centres where diagnostic modalities to invasively assess MVA are available. If this reflects the real clinical practice, a large proportion of patients with microvascular dysfunction might be undiagnosed as in these non-academic or peripheral settings only non-invasive ischaemia testing is used for the diagnosis. Moreover, our results also underscore the need to consider diagnostic modalities assessing impaired microvascular functioning among patients presenting with symptoms and a negative ischaemia test, who have persisting complaints.

Regarding the interpretation of the prevalence of suspected and definitive MVA several factors need to be considered. First, as the international standardized criteria for MVA were published quite recently, various definitions for MVA and non-obstructive CAD were used in the individual studies. This might have led to both under- and overestimation of the true prevalence of MVA in the population. Second, our study focused on specifically MVA among symptomatic patients without coronary obstruction. However, MVA can also occur in other clinical settings,^31^ including in the presence of structural heart diseases. Also, previous studies have suggested that MVA might be an early preclinical sign or manifestation of obstructive CAD^32–34^ and coexist with obstructive epicardial CAD and atherosclerosis.^5^^,^^33^ Additionally, MVA may also be present in other clinical settings including patients across the spectrum of acute coronary syndromes and chronic coronary artery syndrome.^1^ MVA in these settings was not addressed in our study. Furthermore, our study included only studies with symptomatic patients. However, coronary microvascular abnormalities may also occur in asymptomatic patients.^5^^,^^35^ As such, the presence of obstructive CAD or the absence of symptoms does not exclude the presence of impaired microvascular function. Therefore, the prevalence reported by our study might be an underestimation of the true prevalence of MVA in the total population. Finally, some previous studies have reported a higher prevalence of MVA compared to our study. As the calculation of prevalence is dependent on the definition used and selection of the patient population, studies using broader definitions (e.g. including epicardial spasm), using an earlier starting point in the diagnostic pathway or including a selected higher-risk population as the source population are more likely to report higher prevalence’s.^6^^,^^7^ Similarly, not consecutive inclusion of patients, use of different diagnostic pathways and different definitions of the population in the studies have lead to reporting of a wide range of prevalences in observational studies and differences in the prevalences in men and women.^36^

The recently published EAPCI Expert Consensus Document refers to the COVADIS criteria for the clinical diagnosis of MVA.^37^ For the first time, international standardized criteria for MVA were published by the COVADIS Group, presenting two types of MVA; suspected MVA and definitive MVA. COVADIS defines the suspected MVA as the presence of symptoms, no-obstructive CAD and either objective evidence of myocardial ischaemia or evidence of impaired microvascular function. This is in line with our definition of suspected MVA. Definitive MVA based on COVADIS criteria is based on the presence of symptoms, no-obstructive CAD and both objective evidence of myocardial ischaemia and evidence of impaired microvascular functions. This is in line with our definitive MVA.^5^

Nevertheless, it should be noted that for the definite diagnosis of MVA in clinical practice, patients with a positive ischaemia test result and no-obstructive CAD should undergo provocative testing for a final diagnosis, as suggested by the more recently established EAPCI Expert Consensus Document.^37^

However, as these recommendations were published very recently, adherence of the included studies to these recommendations was not possible. As such, also the implementation of these recommendations in current clinical practice remains unknown.

Our categorization is in line with both the COVADIS criteria and the more recently published EAPCI Expert Consensus Document, which similarly suggests use of non-invasive ischaemia tests for ischaemia and no obstructive coronary artery disease patients.^37^

### 4.2 Diagnostic modalities and MVA prevalence

Patients with MVA represent a heterogeneous group and several different pathological mechanisms are implicated in MVA. As various diagnostic modalities assess different aspects of MVA, assessment of one single underlying mechanism of MVA may not be sufficient to diagnose or rule out MVA. Moreover, the currently used non-invasive techniques were originally used to detect ischaemia in obstructive CAD and often do not seem sensitive enough to detect MVA, especially the vasospasm of the microvessels. Nevertheless, by performing an invasive assessment of the microvessels, a comprehensive assessment of multiple underlying mechanisms of MVA is possible, including vasodilatory capacity, coronary microspasm and impaired microvascular resistance. A recent study performing such assessment among a consecutive patient population showed indeed a higher prevalence of MVA (52%) among patients with symptoms and no-obstruction.^38^

Additionally, many studies have assessed CFR in only one vessel. However, it has been shown that microvascular dysfunction can be heterogeneous in the myocardium (patchy distribution).^39^ Therefore, the reported percentages in our study might be an underestimation of the true prevalence and a comprehensive assessment of MVA might yield a higher prevalence.

Although non-invasive and invasive diagnostic modalities are widely accepted and incorporated in the international standardized criteria, the gold standard to diagnose MVA and the accuracy of different non-invasive and invasive diagnostic modalities are still an area of ongoing debate. Due to the suboptimal diagnostic performance of the currently available diagnostic modalities to diagnose MVA, partly caused by the patchy distribution of microvascular disease, a considerable proportion of the patients with MVA might not be diagnosed as such.^40^ This might be another argument for underestimation of the true proportion in patients undergoing these diagnostic tests.

Furthermore, the use of different types of non-invasive stress tests, including exercise treadmill test, stress echo, and stress single-photon-emission computed tomography (SPECT), positron emission tomography (PET), and cardiac magnetic resonance imaging—with different test sensitivities—could lead to underdiagnosis and variation in the reported prevalences, as the prevalence is dependent on the performance of the diagnostic modalities used. The exercise treadmill test is known to have lower sensitivity compared to other imaging modalities including stress echo and stress SPECT, magnetic resonance imaging, and PET.^16^ Moreover, it should also be noted that prior studies among women have shown that non-invasive stress tests may have a lower test sensitivity compared to men. This might suggest that the underestimation of the prevalence may be more pronounced among women.^41^

### 4.3 Clinical characteristics

Overall, no large differences were observed for patients with MVA compared to patients without MVA regarding clinical characteristics. This suggests that other, as yet unidentified, risk factors could account for MVA. However, as these studies have included selected populations, the results might have been influenced by referral bias. Nevertheless, previous studies have also shown that traditional risk factors explain little of the variation of the measures of microvascular function.^7^^,^^42^ This highlights the need to investigate risk factors, other than the traditional cardiovascular risk factors, in future studies.

### 4.4 Sex differences

Although overall more women were included in the studies, leading to higher proportions of female cases, the women-to-men ratio was higher for cases with MVA compared to the total included population. This suggests that the prevalence of MVA is higher in women compared to men.

As prior meta-analyses have shown that no-obstruction is more prevalent in women compared to men, selection bias in favour of women might not be the *only* reason for the high women-to-men ratio in the included studies. However, trials included more women compared to no-trial studies. Also, in this systematic review, most studies assessed MVA using the acetylcholine coronary reactivity test. Therefore, sex differences seem partly related to both the type of MVA assessed and the referral pattern of patients.

While our results indicate that MVA might indeed be more prevalent in women, this may also be dependent on the type of MVA under study. However, no data were available to assess the women-to-men ratio in suspected MVA using non-invasive ischaemia tests and definitive MVA patients. Also, no data were available for MVA assessed by the TFC, as these studies were not eligible for inclusion in this study, while this could be more common among men. Moreover, as obstructive CAD is more prevalent among men^27^^,^^28^ MVA may be more common among men in other clinical settings, e.g. concurrent with obstructive CAD or after successful revascularization, which were out of the scope of this study.

### 4.5 Implications of our findings for clinical care

In this study, we observed substantially high prevalences for different types of MVA, based on diagnostic pathways and modalities. The majority of the studies only assessed one type of underlying mechanism of MVA. Importantly, it should be noted that, when the limitations of these diagnostic modalities (i.e. diagnostic performance, assessment of only one underlying mechanism) are taken into account, the prevalence may be even higher. Moreover, our results for definitive MVA also showed that diagnosis based on only non-invasive ischaemia tests will largely lead to an underdiagnosis of MVA patients. As such, our results suggest that a comprehensive (invasive) evaluation of different underlying mechanisms of MVA is needed to adequately diagnose or rule out MVA in these patients.

This not only has implications for the diagnosis, but also for subsequent therapeutic strategies. In a recent randomized trial, treatment based on the underlying mechanism of MVA improved treatment outcomes including angina severity and quality of life.^38^

This all indicates that modification of the current diagnostic pathways and subsequent clinical care is required to adequately diagnose, manage and treat these substantially large number of patients. Notably, although sex differences were observed in our study, the proportion of men with MVA was also substantial. As such, MVA should also be considered and ruled out in symptomatic men with similar care as in women. However, possible differences in the underlying mechanisms of MVA for men compared to women should be taken into account.

### 4.6 Strength

We used two approaches to assess the prevalence in two clinically relevant populations; (i) among patients with symptoms and a positive non-invasive ischaemia test result (reflecting an estimate for the general cardiology patient population), and (ii) among patients with symptoms and no-obstructive CAD, where additional testing for impaired microvascular function was available (reflecting an estimate for selected patient population referred to specialized centres).

By using a broad definition including the prevalence’s of both suspected and definitive MVA in separate populations, our study provides a complete overview relevant for both community hospitals (e.g. suspected MVA using non-invasive ischaemia tests) and specialized centres (e.g. suspected MVA using specific modalities for MVA and definitive MVA).

### 4.7 Limitations

Although we aimed to include all published studies using a broad search strategy, we cannot exclude that we might have missed some studies. Second, due to the nature of this study, our results are dependent on the available published data and thus susceptible to publication bias and selection bias and restricted to heterogeneity between studies, including suboptimal definitions.

While selection bias could affect both suspected and definitive MVA reported in our study, it may particularly be relevant for suspected MVA using specific modalities for MVA and definitive MVA. Although we only included studies that recruited patients in a consecutive manner, referral bias and selection bias might still exist. This could be more pertinent to the studies investigating the prevalence of suspected MVA using specific modalities for MVA and definitive MVA as these studies are performed in tertiary specialized centres performing additional physiological testing for the assessment of MVA and might therefore include a selected group of patients with the most severe symptoms. Selection of patients with most severe symptoms could have led to a twice as high prevalence compared to patient populations recruited consecutively as shown by the comparison of the studies by Sara *et al*.^7^ and Ong *et al*.^6^ Therefore, the generalizability of the percentages regarding prevalence of suspected MVA using specific modalities for MVA reported in these studies might be restricted to centres with the same characteristics. While selection bias is likely, we believe that this does not substantially affect our conclusion that the prevalence of MVA is large in this population, as we also observed that the prevalence of suspected MVA using non-invasive ischaemia tests was substantial. The diagnostic modalities used to diagnose suspected MVA using non-invasive ischaemia tests, in contrast to suspected MVA using specific modalities for MVA, are widely available in general clinical cardiology practice and therefore, the results can be extrapolated to the general population referred to general cardiology practice.

Heterogeneity between studies included assessment of different types of underlying mechanisms of MVA, including endothelial-dependent and endothelial-independent mechanisms (i.e. microvascular spasm assessed by the acetylcholine coronary reactivity test and CFR) and inclusion of specific populations (including i.e. metabolic syndrome, hypertension, diabetes). This may have lead to over- and underestimation of the MVA in the overall population. Importantly, lack of uniform definitions for both no-obstructive CAD and MVA and the use of different definitions for these two conditions are another source of heterogeneity. As in this systematic review, we did not have access to patient-level data, we could not reclassify data at patient-level according to the recent COVADIS criteria. Diagnosis of suspected and definitive MVA requires a refined and extensive diagnostic strategy. The median prevalences of suspected and definitive MVA were derived from studies including patients who had undergone specific diagnostic pathways. As such, the median percentages reported in our study cannot be used to extrapolate these data to patients or populations who underwent different diagnostic pathways (i.e. patients with no-obstruction with no further patient-level data on the stress test performed and microvascular function). However, as the COVADIS criteria (which was first introduced the definition of suspected and definitive MVA) were published very recently, the patient-level information of NOCAD and MVA using the exact cut-off mentioned in the COVADIS criteria was not available in studies published earlier. Therefore, aggregated data based on the definition in each study were used. Nevertheless, different diagnostic pathways were not aggregated in one definition of MVA.

Moreover, studies assessed only one type of MVA. Therefore, assessment of overlap between different underlying mechanisms was not possible.

Finally, although the inclusion criteria was to have stable symptoms, it should be noted that some studies also included asymptomatic patients (ranging from 3% to 33% of the total population), which may have led to underestimation of the results. Furthermore, heterogeneity in the use of different types of non-invasive stress tests with different test sensitivities in various studies should be mentioned as a limitation, as the prevalence is dependent on the performance of the diagnostic modalities used.

### 4.8 Future perspectives

Our findings indicate that MVA is a common clinical problem among both the general and selected symptomatic patient populations. Although MVA is more prevalent among women compared to men, this might partly be related to referral patterns and the underlying mechanism of MVA.

The results of this study should warrant cardiologists to support, promote and facilitate the routine and comprehensive evaluation of the coronary microcirculation for patients with symptoms and no-obstructive CAD. As a first step, routine invasive assessments immediately after coronary angiograms, including coronary reactivity tests, could be safely performed with low complications, for a comprehensive and adequate diagnosis of different underlying mechanisms of MVA.

## Supplementary material

Supplementary material is available at *Cardiovascular Research* online.

## Supplementary Material

cvab061_Supplementary_Data
