# Supplementary material for: Prevalence of microvascular angina among patients with stable symptoms in the absence of obstructive coronary artery disease: a systematic review
Source: Cardiovasc Res. 2021 Mar 2;118(3):763–71. doi: 10.1093/cvr/cvab061 (PMC8859625; doi:10.1093/cvr/cvab061)
Supplement: cvab061_Supplementary_Data [file cvab061_supplementary_data.pdf]

SUPPLEMENTARY MATERIAL

Prevalence of microvascular angina among patients with stable symptoms in the absence of obstructive coronary artery disease: a systematic review

Elif Aribas[1]*, MD, MSc, Jeanine E. Roeters van Lennep[2]*, MD, PhD, Suzette E. Elias-Smale[3], MD, PhD, Jan J. Piek[4], MD, PhD, Maurits Roos[1], Fariba Ahmadizar[1], PhD, Banafsheh Arshi[1] MD, MSc, Dirk J. Duncker[5], MD, PhD, Yolande Appelman[6], MD, PhD Maryam Kavousi[1], MD, PhD

[1]Department of Epidemiology, Erasmus MC, University Medical Center Rotterdam, Rotterdam, The Netherlands
[2]Department of Internal Medicine, Erasmus MC, University Medical Center, Rotterdam, The Netherlands
[3]Department of Cardiology, Radboud University Medical Center, Nijmegen, The Netherlands
[4]Department of Cardiology, Amsterdam University Medical Centers, location AMC, Amsterdam, The Netherlands
[5]Department of Cardiology, Erasmus MC, University Medical Center Rotterdam, Rotterdam, The Netherlands
[6]Department of Cardiology, Amsterdam University Medical Centers, location VU University Medical Center, Amsterdam, The Netherlands

* denotes equal contribution

CONTENT

Supplementary Methods 1. Search strategy
Supplementary Methods 2. PRISMA checklist
Supplementary Methods 3. PRISMA flow diagram
Supplementary Table 1a. Characteristics of the included studies suspected for MVA using non-invasive ischemia test results
Supplementary Table 1b. Characteristics of the included studies suspected MVA using specific modalities for MVA
Supplementary Table 1c. Characteristics of the included studies for definite MVA
Supplementary Figure 1. Study flowchart

Supplementary Methods 1. Search strategy

(('coronary artery disease'/de OR 'acute coronary syndrome'/exp OR 'coronary artery atherosclerosis'/de OR 'coronary artery calcification'/de OR 'coronary artery constriction'/exp OR 'coronary artery thrombosis'/de OR 'heart infarction'/exp OR 'angina pectoris'/exp OR 'angiocardiography'/de OR 'coronary angiography'/de OR 'coronary artery'/de OR 'coronary blood vessel'/de OR 'ischemic heart disease'/de OR 'heart muscle ischemia'/exp OR 'silent myocardial ischemia'/de OR 'coronary plaque'/de OR 'coronary artery obstruction'/exp OR 'coronary artery blood flow'/de OR (((coronar* OR intracoronar*) NEAR/3 (disease* OR acute OR syndrome* OR atherosclero* OR calcif* OR constrict* OR thrombo* OR angiogra* OR arter* OR vessel* OR plaque* OR Non-obstruct* OR Nonobstruct* OR unobstruct* OR un-obstruct* OR circulat* OR blood-flow*)) OR ((myocard* OR heart) NEAR/3 (infarct* OR angiograph* OR ischem* OR ischaem*)) OR angina OR angiocardiogra*):ab,ti) AND ((Non-obstruct* OR Nonobstruct* OR unobstruct* OR un-obstruct* OR ((no OR absen* OR without OR 'not significan*' OR 'no significan*' OR 'non significan*' OR 'nonsignifican*' OR insignifican*) NEAR/3 obstruct*)):ab,ti) OR (microangiopathy/de OR microvasculature/de OR 'microcirculation'/de OR 'microvascular ischemia'/de OR 'syndrome X'/de OR (microvascul* OR micro-vascul* OR microcircul* OR micro-circul* OR syndrome-X OR X-syndrome):ab,ti) AND ('heart disease'/exp OR heart/exp OR (heart OR cardiac OR myocarc* OR coronar*):ab,ti) AND (('normal coron*' OR 'no stenosis*' OR 'nonculprit*' OR 'without obstruction*' OR 'NOCAD' OR Non-obstruct* OR Nonobstruct* OR unobstruct* OR un-obstruct* OR ((no OR absen* OR without OR 'not significan*' OR 'no significan*' OR 'non significan*' OR 'nonsignifican*' OR insignifican*) NEAR/3 (obstruct* OR steno*)) OR (50 NEAR/3 (stenos* OR obstruct))):ab,ti)) AND (('heart muscle perfusion'/exp OR 'myocardial perfusion imaging'/de OR 'radiodiagnosis'/exp OR 'diagnostic imaging'/de OR 'computer assisted tomography'/exp OR 'stress echocardiography'/de OR (((stress OR perfusion*) NEAR/3 (echocardiogra* OR ecg OR adenosin* OR imag* OR myocard* OR dobutamin*)) OR ((myocard* OR heart-musc*) NEAR/3 (perfusion* OR scintigra*)) OR radiodiagnos* OR mri OR (magnet* NEAR/3 resonan*) OR ((comput* OR positron*) NEAR/3 tomogra*) OR ((ct) NEAR/3 (scan* OR cardiac)) OR spect or PET):ab,ti) OR ('Doppler flowmetry'/exp OR 'ergometrine'/de ('acetylcholine'/de AND 'provocation test'/de) OR ((flow NEAR/3 (velocity*)) OR cfr OR cfvr OR imr OR (index NEAR/6 resistance) OR (TiMI NEAR/3 frame) OR (thromboly* NEAR/6 myocardial* NEAR/6 frame*) OR (Doppler NEAR/3 flowmetr*) OR acetylcholin* OR adenosin* OR ergometrin* OR ergonovin* OR (Provocat* NEAR/3 Spasm)):ab,ti)) NOT ([animals]/lim NOT [humans]/lim) NOT ([Conference Abstract]/lim OR [Letter]/lim OR [Note]/lim OR [Editorial]/lim)

Supplementary Methods 2. PRISMA checklist

Section/topic	#	Checklist item	Reported on page #
**TITLE**			
Title	1	Identify the report as a systematic review, meta-analysis, or both.	1
**ABSTRACT**			
Structured summary	2	Provide a structured summary including, as applicable: background; objectives; data sources; study eligibility criteria, participants, and interventions; study appraisal and synthesis methods; results; limitations; conclusions and implications of key findings; systematic review registration number.	3
**INTRODUCTION**			
Rationale	3	Describe the rationale for the review in the context of what is already known.	4, 5
Objectives	4	Provide an explicit statement of questions being addressed with reference to participants, interventions, comparisons, outcomes, and study design (PICOS).	5, 6
**METHODS**			
Protocol and registration	5	Indicate if a review protocol exists, if and where it can be accessed (e.g., Web address), and, if available, provide registration information including registration number.	5-8
Eligibility criteria	6	Specify study characteristics (e.g., PICOS, length of follow-up) and report characteristics (e.g., years considered, language, publication status) used as criteria for eligibility, giving rationale.	5-8
Information sources	7	Describe all information sources (e.g., databases with dates of coverage, contact with study authors to identify additional studies) in the search and date last searched.	5-8
Search	8	Present full electronic search strategy for at least one database, including any limits used, such that it could be repeated.	Suppl. Material. 2
Study selection	9	State the process for selecting studies (i.e., screening, eligibility, included in systematic review, and, if applicable, included in the meta-analysis).	5-8

Data collection process	10	Describe method of data extraction from reports (e.g., piloted forms, independently, in duplicate) and any processes for obtaining and confirming data from investigators.	7, 8
Data items	11	List and define all variables for which data were sought (e.g., PICOS, funding sources) and any assumptions and simplifications made.	7, 9
Risk of bias in individual studies	12	Describe methods used for assessing risk of bias of individual studies (including specification of whether this was done at the study or outcome level), and how this information is to be used in any data synthesis.	7-8
Summary measures	13	State the principal summary measures (e.g., risk ratio, difference in means).	7-8
Synthesis of results	14	Describe the methods of handling data and combining results of studies, if done, including measures of consistency (e.g., I^2) for each meta-analysis.	7-8
Risk of bias across studies	15	Specify any assessment of risk of bias that may affect the cumulative evidence (e.g., publication bias, selective reporting within studies).	7-8
Additional analyses	16	Describe methods of additional analyses (e.g., sensitivity or subgroup analyses, meta-regression), if done, indicating which were pre-specified.	7-8
**RESULTS**			
Study selection	17	Give numbers of studies screened, assessed for eligibility, and included in the review, with reasons for exclusions at each stage, ideally with a flow diagram.	Suppl. Material, 6, 14
Study characteristics	18	For each study, present characteristics for which data were extracted (e.g., study size, PICOS, follow-up period) and provide the citations.	Suppl. Material 7-11
Risk of bias within studies	19	Present data on risk of bias of each study and, if available, any outcome level assessment (see item 12).	Suppl. Material 7-11
Results of individual studies	20	For all outcomes considered (benefits or harms), present, for each study: (a) simple summary data for each intervention group (b) effect estimates and confidence intervals, ideally with a forest plot.	8-11
Synthesis of results	21	Present results of each meta-analysis done, including confidence intervals and measures of consistency.	

Risk of bias across studies	22	Present results of any assessment of risk of bias across studies (see Item 15).	8-11
Additional analysis	23	Give results of additional analyses, if done (e.g., sensitivity or subgroup analyses, meta-regression [see Item 16]).	8-11
**DISCUSSION**			
Summary of evidence	24	Summarize the main findings including the strength of evidence for each main outcome; consider their relevance to key groups (e.g., healthcare providers, users, and policy makers).	11-22
Limitations	25	Discuss limitations at study and outcome level (e.g., risk of bias), and at review-level (e.g., incomplete retrieval of identified research, reporting bias).	12-17, 20-22
Conclusions	26	Provide a general interpretation of the results in the context of other evidence, and implications for future research.	12-16, 18-19
**FUNDING**			
Funding	27	Describe sources of funding for the systematic review and other support (e.g., supply of data); role of funders for the systematic review.	23

From: Moher D, Liberati A, Tetzlaff J, Altman DG, The PRISMA Group (2009). Preferred Reporting Items for Systematic Reviews and Meta-Analyses: The PRISMA Statement. PLoS Med 6(6): e1000097. doi:10.1371/journal.pmed1000097

For more information, visit: **www.prisma-statement.org**.

Supplementary Methods 3. PRISMA flow diagram

PRISMA 2009 Flow Diagram

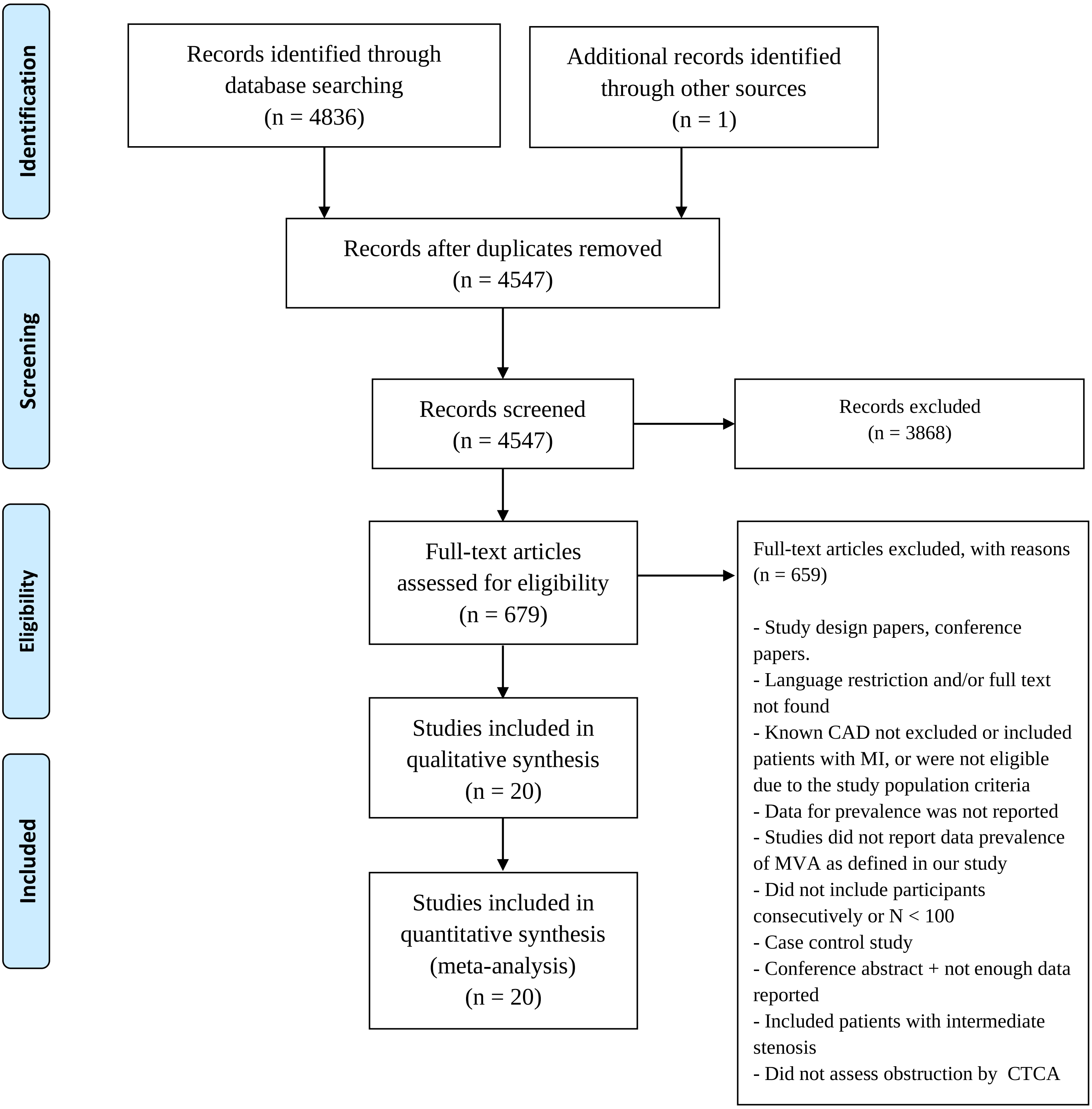

Supplementary Table 1a. Characteristics of the included studies for suspected MVA using non-invasive ischemia test results

**Author, year**	**Study design**	**Country**	**Year interval patients included**	**Total N (stress test performed)**	**Non-invasive stress test modality**	**CTCA or ICA**	**Definition NOCAD**	**Definition positive stress test**	**Prevalence suspected MVA using non-invasive ischemia test results**
Albadarin, 2011 (1)	Database	USA	2001-2003	538 (N positive stress test)	Stress echo	ICA	<50%	The presence of new wall motion abnormalities at peak stress in patients with normal resting images, the development of new WMA at peak stress in the presence of resting WMA, or the presence of unchanged WMA both at rest and peak stress.	43%
Cheezum, 2015 (2)	Data registry	USA	2005-2011	417	ETT	CTCA	<50%	Upsloping ST depressions ≥1.5, or down sloping or horizontal depressions ≥1.0 mm in at least two leads.	53%
Chinnaiyan, 2012 (3)	Prospective registry	USA	NR	6198	SPECT MPI, stress echo or ETT	CTCA	<50%	Decided by referring physician.	37%
Fragasso, 1999 (4)	Prospective trial	Italy	NR	101 (N positive stress test)	ETT	ICA	<50%	NR	44%
Groothuis, 2010 (5)	Prospective trial	The Netherlands	NR	154	MRI MPI	CTCA	<50%	Hypoperfusion during more than three consecutive images after arrival of the contrast agent in the left ventricular cavity.	33%
Hachamov, 2012 (6)	Prospective registry	USA	NR	1703	SPECT/PET MPI	CTCA	<70% for a major epicardial coronary artery or <50% for the left main coronary artery.	NR	34%
Lin, 2012 (7)	Prospective registry	USA	1991-2007	9941	ETT	ICA	<70% for a major epicardial coronary artery or <50% for the left main coronary artery.	>=1mm stress induced ST depression in the absence of baseline ST-T changes.	65%

Patel, 2014 (8)	Prospective registry	USA	2009-2011	423080 (N stress test performed)	Stress SPECT MPI, stress echo, ETT or stress CMR	ICA	<50% in all major epicardial or branch vessels of 2.0 mm in diameter.	NR	53%
Schuijf, 2006 (9)	Prospective trial	The Netherlands	NR	74	SPECT MPI	CTCA	<50%	Hypoperfusion during more than three consecutive images after arrival of the contrast agent in the left ventricular cavity.	35%

MVA; microvascular angina, N; number, CTCA; computed tomography coronary angiography, ICA; invasive coronary angiography, NOCAD; non-obstructive coronary artery disease, MRI; magnetic resonance imaging, MPI; myocardial perfusion imaging, SPECT; single-photon emission computed tomography, ETT; exercise treadmill test, echo; echocardiogram, NR; not reported.

Supplementary Table 1b. Characteristics of the included studies for suspected MVA using specific modalities for MVA

Author, year	Country	Year interval patients included	Total N (NOCAD)	Trial or No-trial	Diagnostic modality used		Definition used		Prevalence MVA using specific modalities for MVA	Description population	Remarks
					CTCA or ICA	Diagnostic modality used for diagnosing MVA	NOCAD	MVA using specific modalities for MVA			
Aziz, 2017 (10)	Germany	2007 - 2014	1379	No-trial	ICA	Acetylcholine test	<50%	If no significant epicardial spasm (>75%) was produced, but a reproduction of the usual symptoms was achieved together with ischemic ECG changes, the test was interpreted as CMD due to microvascular spasm.	33.2%	Patients with stable angina and noobstruction on coronary angiography.	
Jespersen, 2014 (11)	Denmark	1999-2009	381	No-trial	ICA	TFC	<50%	CTFC >28	27.8%	Patients with symptoms and no-obstruction, on coronary angiography.	Nested-case control study, however, the prevalence among no cases is almost the same, and all participants were consecutively included.
Nemes, 2007 (12)	Hungary	1997-2004	119	Trial	ICA	TTDE CFR	<50%	CFR <1.73	25.2%	Patients with stable or atypical angina referred for coronaryangiography with no-obstruction.	
Odaka, 2017 (13)	Japan	2011-2014	198	No-trial	ICA	Acetylcholine test	<50%	Myocardial lactate production without epicardial spasm.	15.2%	Patients with chest pain and/or electrocardiography abnormalities and no-obstruction on coronaryangiography.	
Ohba, 2012 (14)	Japan	2002-2011	370	Trial	ICA	Acetylcholine test	<50%	Positive for lactate production and a decrease in quantitative coronary blood flow without epicardial vasospasm, associated with the occurrence of chest symptoms and ischemic changes in the electrocardiogram.	13.5%	Stable patients with suspected angina and no-obstruction on coronaryangiography.	

Ong, 2012 (15)	Germany	2007-2008	144	Trial	ICA	Acetylcholine test	<20%	Reproduction of symptoms, ischemic electrocardiographic changes, and no epicardial spasm.	29.6%	Patients with stable, exertional-related angina and no-obstruction on coronary angiography.	
Petersen, 2014 (16)	USA	NR	298	Trial	ICA	TFC	<50%	TFC >=35	39.9%	Patients with symptoms and signs of ischemia and no-obstruction on coronary angiography.	
Reis, 2001 (17)	USA	NR	159	Trial	IVA	IV CFVR	<50%	CFVR <2.5	46.5%	Patients with chest pain and no-obstruction on coronary angiography.	
Schoenenberger, 2016 (18)	Switzerla nd	1997 - 2008	707	Trial	ICA	Acetylcholine test	<50%	If there was no relevant coronary diameter change (<50% reduction of lumen diameter) after acetylcholine, but there were typical symptoms after acetylcholine, during contrast media application, during atrial pacing, during femoral puncture and/or during extra beats.	40.0%	Patients with angina equivalent symptoms and no coronary stenosis.	
Sicari, 2009 (19)	Italy	2002-2007	394	No-trial	ICA	TTDE CFR	<50%	CFR <2.0	22.1%	Patients with chest pain and normal coronary angiography	
Sun, 2005 (20)	Japan	1995-2000	131	No-trial	ICA	Acetylcholine test	Normal coronary arteriogram s	When intracoronary acetylcholine induced myocardial ischemia (2 or more signs out of chest pain, ischemic ECG changes, and myocardial lactate production).	26.7%	Patients with chest pain and normal coronary angiography	

MVA; microvascular angina, N; number, CTCA; computed tomography coronary angiography, ICA; invasive coronary angiography, NOCAD; non-obstructive coronary artery disease, NR; not reported, (C)TFC; (corrected) TIMI frame count, ECG; electrocardiography, TTDE; transthoracic doppler echocardiography, CFVR; coronary flow velocity reserve, IV; invasive, CFR; coronary flow reserve, CBF; coronary blood flow, PCI; percutane coronary intervention.

Supplementary Table 1c. Characteristics of the included studies for definite MVA

Author, year	Total N (patients with NOCAD)	N cases with CMD type B	N cases with diagnosed with CMD type B who had also undergone ischemia testing	Prevalence CMD type C (%)	Test performed for CMD type B	Non-invasive stress test performed	Proportion Ischemia test performed (%)	Remarks
Reis, 2001 (17)	159	74	59	22/59 (37.3%)	IV CFVR	Stress ECG, stress echocardiography or MPI	79.8%	
Schoenenberger, 2016 (18)	707	283	208	39/208 (18.8%)	Acetylcholine test	ETT	73.5%	
Schoenenberger, 2016 (18)	707	283	209	43/209 (20.6%)	Acetylcholine test	Stress echo	73.8%	
Ohba, 2012 (14)	370	50	41	9/41 (22.0%)	Acetylcholine test	ETT	82%	
Ohba, 2012 (14)	370	50	32	8/32 (25.0%)	Acetylcholine test	Stress SPECT	62.8%	
Ong, 2012 (15)	144	42	35	21/35 (60.0%)	Acetylcholine test	Non-invasive test for ischemia *(not further specified)*	83%	

MVA; microvascular angina, NOCAD; non-obstructive coronary artery disease, NR; not reported, TFC; TIMI frame count, IV CFVR; invasive coronary flow velocity reserve, ETT; exercise treadmill test, echo; echocardiogram, MPI; myocardial perfusion imaging, SPECT; single-photon emission computed tomography.

References

1. Al Badarin FJ, From AM, McCully RB, Lopez-Jimenez F. Likelihood of obstructive coronary disease in metabolic syndrome patients with abnormal stress echocardiography. Int J Cardiol. 2011;152(2):207-11.
2. Cheezum MK, Subramaniyam P, Bittencourt MS, Hulten EA, Ghoshhajra BB, Shah NR, et al. Prognostic value of coronary CTAvs. exercise treadmilltesting: Results fromthePartnersregistry. Eur Heart J Cardiovasc Imaging. 2015;16(12):1338-46.
3. Chinnaiyan KM, Raff GL, Goraya T, Ananthasubramaniam K, Gallagher MJ, Abidov A, et al. Coronary computed tomography angiography after stress testing: results from a multicenter, statewide registry, ACIC (Advanced Cardiovascular Imaging Consortium). Journal of the american college of cardiology. 2012;59(7):688-95.
4. Fragasso G, Lu C, Dabrowski P, Pagnotta P, Sheiban I, Chierchia SL. Comparison of stress/rest myocardial perfusion tomography, dipyridamole and dobutamine stress echocardiography for the detection of coronary disease in hypertensive patients with chest pain and positive exercise test. J Am Coll Cardiol. 1999;34(2):441-7.
5. Groothuis JGJ, Beek AM, Brinckman SL, Meijerink MR, Koestner SC, Nijveldt R, et al. Low to intermediate probability of coronary artery disease: Comparison of coronary CT angiography with first-pass MR myocardial perfusion imaging. Radiology. 2010;254(2):384-92.
6. Hachamovitch R, Nutter B, Hlatky MA, Shaw LJ, Ridner ML, Dorbala S, et al. Patient management after noninvasive cardiac imaging: Results from SPARC (Study of myocardial perfusion and coronary anatomy imaging roles in coronary artery disease). J Am Coll Cardiol. 2012;59(5):462-74.
7. Lin T, Rechenmacher S, Rasool S, Varadarajan P, Pai RG. Reduced survival in patients with "coronary microvascular diseaseo". Int J Angiol. 2012;21(2):89-94.
8. Patel MR, Dai D, Hernandez AF, Douglas PS, Messenger J, Garratt KN, et al. Prevalence and predictors of nonobstructive coronary artery disease identified with coronary angiography in contemporary clinical practice. Am Heart J. 2014;167(6):846-52.e2.
9. Schuijf JD, Wijns W, Jukema JW, Atsma DE, de Roos A, Lamb HJ, et al. Relationship Between Noninvasive Coronary Angiography With Multi-Slice Computed Tomography and Myocardial Perfusion Imaging. J Am Coll Cardiol. 2006;48(12):2508-14.
10. Aziz A, Hansen HS, Sechtem U, Prescott E, Ong P. Sex-Related Differences in Vasomotor Function in Patients With Angina and Unobstructed Coronary Arteries. J Am Coll Cardiol. 2017;70(19):2349-58.
11. Jespersen L, Abildstrøm SZ, Peña A, Hansen PR, Prescott E. Predictive value of the corrected TIMI frame count in patients with suspected angina pectoris but no obstructive coronary artery disease at angiography. Clin Res Cardiol. 2014;103(5):381-7.
12. Nemes A, Forster T, Geleijnse ML, Kutyifa V, Neu K, Soliman OII, et al. The additional prognostic power of diabetes mellitus on coronary flow reserve in patients with suspected coronary artery disease. Diabetes Res Clin Pract. 2007;78(1):126-31.
13. Odaka Y, Takahashi J, Tsuburaya R, Nishimiya K, Hao K, Matsumoto Y, et al. Plasma concentration of serotonin is a novel biomarker for coronarymicrovascular dysfunction in patients with suspected angina and unobstructive coronary arteries. Eur Heart J. 2017;38(7):489-96.
14. Ohba K, Sugiyama S, Sumida H, Nozaki T, Matsubara J, Matsuzawa Y, et al. Microvascular coronary artery spasm presents distinctive clinical features with endothelial dysfunction as nonobstructive coronary artery disease. J Am Heart Assoc. 2012;1(5).
15. Ong P, Athanasiadis A, Borgulya G, Mahrholdt H, Kaski JC, Sechtem U. High prevalence of a pathological response to acetylcholine testing in patients with stable angina

pectoris and unobstructed coronary arteries. The ACOVA Study (Abnormal COronary VAsomotion in patients with stable angina and unobstructed coronary arteries). J Am Coll Cardiol. 2012;59(7):655-62.
16. Petersen JW, Johnson BD, Kip KE, Anderson RD, Handberg EM, Sharaf B, et al. TIMI frame count and adverse events in women with no obstructive coronary disease: A pilot study from the NHLBI-sponsored Women's Ischemia Syndrome Evaluation (WISE). PLoS ONE. 2014;9(5).
17. Reis SE, Holubkov R, Conrad Smith AJ, Kelsey SF, Sharaf BL, Reichek N, et al. Coronary microvascular dysfunction is highly prevalent in women with chest pain in the absence of coronary artery disease: results from the NHLBI WISE study. Am Heart J. 2001;141(5):735-41.
18. Schoenenberger AW, Adler E, Gujer S, Jamshidi P, Kobza R, Stuck AE, et al. Prognostic value of an abnormal response to acetylcholine in patients with angina and non-obstructive coronary artery disease: Long-term follow-up of the Heart Quest cohort. Int J Cardiol. 2016;221:539-45.
19. Sicari R, Rigo F, Cortigiani L, Gherardi S, Galderisi M, Picano E. Additive Prognostic Value of Coronary Flow Reserve in Patients With Chest Pain Syndrome and Normal or Near-Normal Coronary Arteries. The American Journal of Cardiology. 2009;103(5):626-31.
20. Sun H, Fukumoto Y, Ito A, Shimokawa H, Sunagawa K. Coronary microvascular dysfunction in patients with microvascular angina: Analysis by TIMI frame count. J Cardiovasc Pharmacol. 2005;46(5):622-6.

Supplementary Figure 1. Study flowchart

N; number, CAD: coronary artery disease, MI; myocardial infarction, MVA; microvascular angina, CTCA; computed tomography coronary angiography, ICA; invasive coronary angiography, MPI; myocardial perfusion imaging, CAC; coronary artery calcium

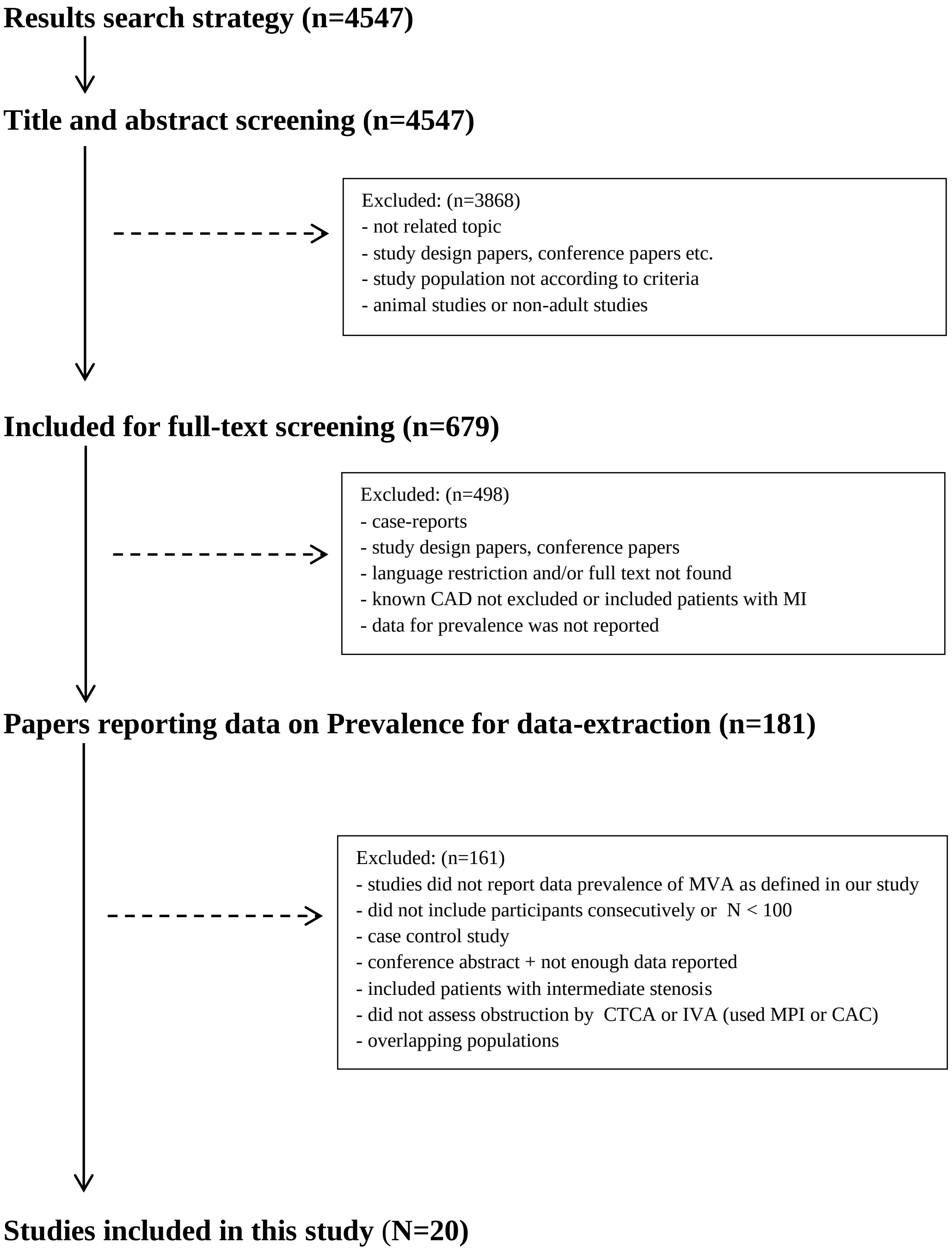

N; number, CAD: coronary artery disease, MI; myocardial infarction, MVA; microvascular angina, CTCA; computed tomography coronary angiography, ICA; invasive coronary angiography, MPI; myocardial perfusion imaging, CAC; coronary artery calcium
